# Diverse social media experiences and adolescents’ depressive symptoms: the moderating role of neurobiological responsivity to rejected peers

**DOI:** 10.1093/scan/nsae070

**Published:** 2024-10-17

**Authors:** Jolien Trekels, Maria T Maza, Jimmy Capella, Nathan A Jorgensen, Seh-Joo Kwon, Kristen A Lindquist, Mitchell J Prinstein, Eva H Telzer

**Affiliations:** Department of Psychology and Neuroscience, University of North Carolina at Chapel Hill, Chapel Hill, United States; Department of Psychology and Neuroscience, University of North Carolina at Chapel Hill, Chapel Hill, United States; Department of Psychology and Neuroscience, University of North Carolina at Chapel Hill, Chapel Hill, United States; Department of Psychology and Neuroscience, University of North Carolina at Chapel Hill, Chapel Hill, United States; Department of Psychology, Rutgers University, Newark, United States; Department of Psychology and Neuroscience, University of North Carolina at Chapel Hill, Chapel Hill, United States; Department of Psychology and Neuroscience, University of North Carolina at Chapel Hill, Chapel Hill, United States; Department of Psychology and Neuroscience, University of North Carolina at Chapel Hill, Chapel Hill, United States

**Keywords:** social media experiences, adolescents, neurobiological sensitivities, depressive symptoms

## Abstract

Adolescents’ experiences with social media are complex and can impact their mental well-being differently. Our study aimed to understand how neurobiological sensitivities may moderate the association between different social media experiences and depressive symptoms. In a multiwave study, 80 adolescents (M_age_ = 13.06, SD = 0.58) took part in an functional magnetic resonance imaging task designed to gauge the neural responses when viewing accepted and rejected peers within their own social networks (Wave 1). We also collected self-reported measures of positive (digital social connection) and negative (digital pressure) experiences on social media and depressive symptoms (Waves 2 and 3). Our findings revealed that there were no significant associations between digital social connection, digital pressure, and depressive symptoms 1 year later. However, the association between digital social connection and depressive symptoms was moderated by neural responsivity. Specifically, for adolescents with reduced sensitivity to their rejected peers in the ventral striatum, right temporoparietal junction, and ventromedial prefrontal cortex, digital social connection was associated with reduced depressive symptoms 1 year later. These results emphasize the importance of individual differences in how adolescents’ brains respond to rejected peers in shaping the impact of online experiences on their mental well-being.

## Introduction

Contemporary youth are growing up in a digitalized environment. Social media platforms provide abundant and distinct opportunities to find social connection, particularly for adolescents whose psychological well-being and social functioning heavily rely on a sense of belonging to a group (Arslan et al. 2020, Smith et al. 2021). Indeed, one of the primary motivations driving adolescents to engage with social media is the desire for social connection (Allen et al. 2014). However, the diverse array of potential interactions with these platforms brings forth varying implications for adolescents’ mental well-being (Choukas-Bradley et al. 2023). Adolescents themselves have indicated they experience different or even opposite affective responses to their social media use, including connection as well as loneliness (Van Der Wal et al. 2024). Scholars, therefore, advocate for research that investigates specific social media experiences that could exacerbate or alleviate negative mental health (Choukas-Bradley et al. 2023) as well as account for individual susceptibilities to better understand how adolescents engage with and are affected by these platforms (Valkenburg et al. 2022a). The present study therefore (I) examines specific social media experiences by distinguishing between different affective responses to social media use (i.e. feeling social pressure and stress versus feeling socially connected) and (II) explores neural susceptibilities that could shed light on which adolescents might benefit from social media and which might be adversely affected.

### Social media experiences and depressive symptoms

Adolescence is an important time for forming enduring, meaningful friendships and gaining acceptance within valued peer groups (Brown 2004). In today’s society, this quest for connection often unfolds through social media platforms, whose unique affordances have significantly transformed adolescents’ interactions with their peers, introducing both positive and negative elements (Nesi et al. 2018). Notably, social media’s constant connectivity enables adolescents to access social connections at any moment, fostering a sense of belonging and reducing feelings of isolation (Verduyn et al. 2017). However, the absence of such desired interactions (e.g. ostracism or being ignored or excluded by others; Williams 2009, Yue et al. 2022), or the pressure to meet social media demands—being constantly available and worrying about peer approval—have been associated with increased depressive symptoms (Nick et al. 2022). Consequently, social media can be both a space for connection and a source of “stress in the service of connection” (Weinstein and Selman 2016, p. 394), where adolescents experience pressure to maintain closeness in a digital space. This duality in adolescents’ experiences and emotional responses to social media use underscores the need for researchers to differentiate between various interactions and experiences on these platforms (Choukas-Bradley et al. 2023).

### Moderation by neurobiological sensitivity to rejected and accepted peers

Adolescence marks a critical neurodevelopmental period during which socio-cognitive skills, such as mentalizing and abstract thinking, are maturing (Blakemore and Mills 2014). These skills allow adolescents to navigate their social environments and form meaningful peer relationships (Güroğlu 2021). At the same time, social cues, including those related to social status such as peer acceptance and rejection, tend to elicit stronger affective responses in adolescents compared to children and adults (Crone and Dahl 2012, Pfeifer and Blakemore 2012, Blakemore and Mills 2014, Güroğlu 2021). Importantly, the significance of peer acceptance and rejection is particularly salient within social media platforms, where the value of social status is frequently showcased through quantifiable feedback metrics that show whether a peer is accepted or rejected (i.e. likes, followers, and comments).

Importantly, not all adolescents thrive in positive social media contexts or suffer in negative ones (Orben 2020). Hence, it is vital to consider individual difference factors, particularly neurobiological sensitivities, which may determine the extent to which social media use influences mental health outcomes during adolescence (Prinstein et al. 2020). Following the Differential Susceptibility to Environmental Influences theory (Belsky et al. 2007) and the Biological Sensitivity to Context theory (Ellis and Boyce 2008), neurobiological sensitivities can shape individuals’ responses to a range of psychosocial experiences, including those encountered online. Notably, these theories underscore adolescence as a period of heightened social salience. Understanding which adolescents are most neurobiologically sensitive may help shed light on which adolescents thrive or suffer in different social contexts (Schriber and Guyer 2016).

Since many peer interactions now occur online, these theories may have important implications for understanding individual differences in how social media use affects youth. Specifically, individuals characterized by heightened environmental susceptibility are assumed to display an enhanced sensitivity to both negative and positive environments (Ellis et al. 2011). Considering that adolescents encounter both positive and negative experiences online (Van Der Wal et al. 2024), increased neurobiological sensitivity may intensify the impact of such experiences on adolescent mental health, for better (when feeling connected and supported on social media) or for worse (when feeling stressed and pressured on social media). Therefore, the aim of the current study is to examine whether neurobiological sensitivity to social status moderates the association between positive (e.g. feeling connected) and negative (e.g. feeling pressured) experiences online and adolescents’ depressive symptoms.

Prior neuroscientific research indicates adolescents’ heightened focus on social hierarchies (Koski et al. 2015), through increased activation in brain regions associated with reward valuation, mentalizing, and social cognition. Specifically, the ventral striatum (VS) is involved in assessing the motivational significance of visual stimuli (Lindquist et al. 2016) and the affective value of peers (Do et al. 2020). Subregions of the prefrontal cortex (PFC), including the ventromedial prefrontal cortex (vmPFC), are activated during the formation of social hierarchies (Wang et al. 2014), with the vmPFC being implicated in the socio-emotional processing and responding to the social status of others (e.g. Cloutier et al. 2012). Additionally, the temporoparietal junction (TPJ) is activated when inferring the mental states and intentions of social members, enabling individuals to process and comprehend rejection (Sahi and Eisenberger 2021), and has been implicated in determining peer status (Schmälzle et al. 2017). Neural responsivity to social status (i.e. peers who are rejected and accepted) in the VS, vmPFC, and TPJ may act as a susceptibility factor in the context of social media experiences and well-being. Consequently, investigating whether heightened neurobiological sensitivity in these regions may determine the extent to which social media impacts adolescents’ depressive symptoms is warranted.

### Current study

The present study examines adolescents’ self-reported positive experiences on social media, specifically relating to feeling socially connected and supported online, as well as adolescents’ self-reported negative experiences, specifically digital pressure regarding availability and approval of peers. Previous research has found that feeling socially supported through online connections decreases depression-related outcomes over time (e.g. Cole et al. 2019), whereas digital stress has been associated with increased depressive symptoms (e.g. Nick et al. 2022). We hypothesized that feeling connected online would be related to decreased levels of depressive symptoms at a 1-year follow-up (H1) and feeling digital pressure on social media would be related to increased depressive symptoms at a 1-year follow-up (H2).

Using a novel, ecologically valid functional magnetic resonance imaging (fMRI) task designed to measure the neural correlates of viewing social status (i.e. accepted or rejected peers) within adolescents’ real-life social networks, we examined whether adolescents’ neural responsivity to peers who are the most accepted and most rejected moderates the links between positive (digital social connection) and negative (digital pressure) experiences and depressive symptoms 1 year later. We focused on brain regions associated with reward valuation (i.e. vmPFC and VS) and social cognition (i.e. rTPJ) given their association with the processing of social information, including both acceptance and rejection (Somerville et al. 2011, Pfeifer and Blakemore 2012, Perino et al. 2016). Since individual susceptibility to the environment may be adaptive or maladaptive, depending on the social context (Ellis et al. 2011), we hypothesized that individuals who show greater neural responsivity to their accepted and rejected peers in these Regions of Interest (ROIs) would have a stronger association between their social media experiences and depressive symptoms (i.e. digital social connection relates to less depressive symptoms [H3a], whereas digital pressure relates to more depressive symptoms, [H3b]). In contrast, adolescents with low neural responsivity to their accepted/rejected peers would show a weaker or null association between digital social connection a digital pressure and depressive symptoms (H4). Sensitivity to both high (i.e. accepted peers) and low (i.e. rejected peers) social status is hypothesized to be a susceptibility marker due to adolescents’ heightened responsiveness to social cues (Schriber and Guyer 2016) and the amplified visibility of rejection and acceptance on social media.

## Material and methods

### Participants

Data for the current study used data collected across three waves when participants were in 7th and 8th grade (Wave 2, 2017–18 academic year), 8th and 9th grade (Wave 3, 2018–19 academic year), and 9th and 10th grade (Wave 4, 2019–20 academic year). For the purposes of this study, demographic information and sociometric nominations were obtained during school-based testing at Wave 2, fMRI data were collected at Wave 2, and self-report measures were collected at Waves 3 and 4. Wave 1 data were omitted from the analysis as none of the measures were included during that wave. All study procedures were approved by the University’s institutional review board and adolescents and their parents provided written assent and consent.

At the second wave, adolescents completed the Classmates task during an fMRI scan. The final analytical sample, which included adolescents with fMRI data who filled out both surveys at Waves 3 and 4, was composed of 80 participants (53.8% biological female, M_age_ = 13.06; SD_age_ = 0.58; age range = 12–14; 26.3% Latinx/Hispanic, 30% Black, 37.5% White, 12.5% multiracial, 5% Native American; see Table 1). Table 1 displays the demographic composition of the analytical sample.

**Table 1.** Demographic composition of the analytical sample

	*N* = 80 in analyses	
Demographic variables	*n*	%
Age		
Mean 13.51		
SD 0.52		
Range 12–14		
Biological sex		
Female	43	54
Male	37	46
Racial identity		
White	24	30
Black	20	25
Hispanic/Latinx	24	30
Multiracial	10	12.5
Other	2	2.5
Family total annual income		
$0–$14 999	9	11.3
$15 000–$29 999	18	22.5
$30 000–$44 999	15	18.8
$45 000–$59 999	18	22.5
$60 000–$74 999	7	8.8
$75 000–$89 999	2	2.5
$90 000–$99 999	3	3.8
$100 000–$119 999	1	1.3
$120 000–$150 000	2	2.5
$150 000 +	2	2.5
NA	3	3.8
Maternal education*		
Less than 8th grade	9	11.39
8th grade completed	1	1.27
Some high school	7	8.86
High school	10	12.66
Some college	27	34.18
Associate’s degree	11	13.92
Bachelor’s degree	5	6.33
Master’s degree (MA, MS)	2	2.53
Professional degree (MD, PhD, etc.)	1	1.27
NA/missing	6	7.59

Demographics collected at Wave 2.

### Measures

#### Classmates task

As part of the 5-year longitudinal study, participants completed a sociometric survey in their classrooms at school to assess peer social status within their own school networks. A total of 873 6th–7th graders were provided with an alphabetized roster of all classmates in their school and grade, counterbalanced A-Z or Z-A and were instructed to select: (I) who they liked the most, (II) who they liked the least, (III) who they perceived as the most popular, and (IV) who they perceived as the least popular. There were no restrictions on the number of peers they could nominate for each category. A standardized difference score between standardized (i.e. within grade) “liked-most” and “liked-least” nominations was calculated to yield a social preference score (i.e. peer acceptance/rejection), with higher scores indicating greater peer acceptance and lower scores indicating greater peer rejection. A similar difference score was calculated for popularity. The data collected from peer nominations during Wave 1 were used to create the stimuli for the subsequent Classmates task, conducted during Wave 2. Additionally, peer nominations were again collected during Wave 2, allowing us to account for the participants’ own likability when conducting our analyses. Social preference scores were highly stable across Waves 1 and 2 of the study (“liked most”: r = 0.80, *P* < .001; “liked least”: r = 0.81, *P* < .001).

During Wave 2, a subset of participants from the larger longitudinal study completed the Classmates task (adapted from Zerubavel et al. 2015, Parkinson et al. 2017) during fMRI. Participants viewed yearbook photos of peers from their school. Yearbook photos (i.e. targets) used in the task were selected based on the sociometric data from the previous year (Wave 1). Peers selected as target images for the task had to have a sociometric *z*-score between 1 and 5 (representing 1–5 SD above the mean on acceptance/rejection or popularity in their school and grade) or between −1 and −5 (representing 1–5 SD below the mean on acceptance/rejection or popularity in their school and grade). Given the school- and grade-specific target images, a version of the task was created for each grade level within each school for a total of six versions. The task had four conditions: High peer acceptance (i.e. *z*-score between 1 and 5 on social preference), high peer rejection (i.e. *z*-score between −1 and −5 on social preference), high popularity (i.e. *z*-score between 1 and 5 on popularity), and low popularity (i.e. *z*-score between −1 and −5 on popularity). [While there is some congruency between popularity and likability, these have been shown to be distinct sociometric constructs and are only moderately correlated among adolescent samples (Cillessen and Mayeux 2004). In our own study, we found that the correlation between social preference and popularity scores ranged from 0.36 to 0.68 within each version of the task (Version 1: r = 0.36, *P* < .05; Version 2: r = 0.68, *P* < .001; Version 3: r = 0.56, *P* < .001; Version 4: r = 0.42, *P* < .01, Version 5: r = 0.46, *P* < .005). We ensured there was no overlap across conditions, such that each target belonged to only one sociometric category and appeared in only one condition. Further, we ensured that peers selected for one category (e.g. high popularity) did not have very high or very low sociometric scores in another category (e.g. over 1SD above or below the mean) helping to ensure the four conditions were as distinct and non-overlapping as possible.] In each condition, participants saw 10 target images, with roughly an equal number of boys and girls. The absolute value of the average *z*-score within each condition was approximately 2 across all versions of the task. Each target image appeared in one condition to avoid any overlap between conditions. The study participants were excluded as stimuli so they would not see their own image. Participants were not explicitly told they would be viewing peers who were rated as the most accepted or most rejected in their grade, as research has shown that adolescents spontaneously track the status of their peers without social cues specifically directing their attention toward their peers’ status (Dai et al. 2023). Instead, they were told they would see yearbook pictures of their classmates and would be playing a task in which they were to press a button if they saw a picture stimulus repeated twice in a row. While the N-back task instruction was intended to serve as an attention check, the real aim of the task was to explore adolescents’ neural responses to passively viewing their peers’ stimuli as has been done in prior tasks (Zerubavel et al. 2015, Parkinson et al. 2017). Target images were collected from the previous year’s school yearbook picture, which was the same year sociometric ratings were collected. All yearbook photos were digitized into JPEG images.

The task was programmed in E-Prime and presented across two runs. Each run consisted of 8 blocks, 2 blocks per condition, each with 10 faces. The eight blocks were presented in a randomized order in each run. The order in which the target images were shown was fixed within each block and pre-selected based on a randomization algorithm. To ensure participants were paying attention, the task had a built-in N-back design such that each block contained one stimulus that appeared twice in a row (Parkinson et al. 2017). Button presses were monitored, and on average participants accurately pressed on 95% of the trials (range: 63%–100%). Thus, no participants were excluded based on noncompliance with the attention checks during the task. Participants were asked to press with their right pointer finger when they saw the target image repeated to ensure they were paying attention. Repeated targets were fixed in the task and balanced so that no target was shown more than another (i.e. if a target was seen twice in one block, it would be absent from the next block). Participants saw each face four times total (twice in each run), with each condition having 40 trials each. Stimuli were presented for 1750 ms and separated by a jittered inter-trial interval (M = 2300 ms).

#### Digital social connection

At Wave 3, adolescents’ positive experiences on social media were measured using a subset of five items adapted from previous national surveys from the Pew Research Center (Anderson and Jiang, 2018), Common Sense Media (Rideout and Robb 2018), and HopeLab (Rideout and Fox 2018). Using a five-point Likert scale, ranging from “1 = Never to 5 = Always,” participants indicated how frequently they experienced social connection on social media (e.g. “I feel supported and encouraged by my friends,” “I feel more connected with my friends,” and “I feel happy because of a positive comment from someone”). The scale showed good internal consistency with α = 0.88. A mean score was calculated with higher scores indicating more digital social connection.

#### Digital pressure

At Wave 3, adolescents’ digital pressure was measured with seven items about their subjective feelings of pressure and distress stemming from availability and peer approval demands on social media. Using a five-point Likert scale, ranging from “1 = Never to 5 = Always,” participants indicated how frequently they experienced digital pressure (e.g. “I feel pressure to quickly respond or ‘like’ my closest friends’ posts,” “I feel pressure to show the best version of myself,” and “I feel pressure to reach a certain number of likes or comments on a post”). The scale showed good internal consistency with α = 0.91. A mean score was calculated with higher scores indicating more digital pressure.

#### Depressive symptoms

Depressive symptoms were evaluated at Wave 3 and Wave 4 using the Short Mood and Feelings Questionnaire (Angold et al. 1995). Participants responded to nine questions to indicate the degree to which they had experienced depressive symptoms over the prior 2 weeks, on a 3-point Likert scale from “0 = not at all true to 2 = mostly true.” A mean score was calculated; higher numbers indicated higher levels of depressive symptoms (Wave 3, α = 0.93; Wave 4, α = 0.92).

#### Covariates

At Wave 3, one item queried participants how often they check social media (Instagram, Facebook, Snapchat) on a 9-point Likert scale ranging from “1 = Almost constantly to 9 = Never.” Frequency of use may confound the association between specific social media experiences and depressive symptoms, so controlling for frequency improves the internal validity of the study by accounting for an important aspect of social media behavior that could otherwise skew results, as demonstrated in numerous other studies (Nesi and Prinstein 2015, Shensa et al. 2017). Participants also indicated their biological sex (1 = male, 2 = female) and age at the time of the scan. Lastly, we measured SES by asking parents to indicate their level of income ranging from 1 = $0–$14.999 to 10 = +$150.000. These measures were used as a covariate in the analyses.

### MRI data acquisition and preprocessing

Imaging data were collected using a 3 Tesla Siemens Prisma MRI scanner. The Classmates Task was presented on a computer screen and projected through a mirror. A high-resolution structural T2*-weighted echo-planar imaging (EPI) volume (TR = 2000 ms; TE = 25 ms; matrix = 92 × 92; FOV = 230 mm; 37 slices; slice thickness = 3 mm; voxel size 2.5 × 2.5 × 3 mm^3^) was acquired coplanar with a T2*-weighted structural matched-bandwidth (MBW), high-resolution, anatomical scan (TR = 5700 ms; TE = 65 ms; matrix = 192 × 192; FOV = 230 mm; 38 slices; slice thickness = 3 mm). In addition, a T1* magnetization-prepared rapid-acquisition gradient echo (TR = 2400 ms; TE = 2.22 ms; matrix = 256 × 256; FOV = 256 mm; sagittal plane; slice thickness = 0.8 mm; 208 slices) was acquired. The orientation for the EPI and MBW scans was oblique axial to maximize brain coverage and to reduce signal dropout.

Preprocessing was conducted using FSL (FMRIB’s Software Library, version 6.0; www.fmrib.ox.ac.uk/fsl) and included the following steps: Skull stripping using Brain Extraction Tool (Smith 2002); motion correction with Method Correction using FMRIB’s (Oxford Center for Functional Magnetic Resonance Imaging of the Brain) Linear Image Registration Tool (Jenkinson et al. 2002); spatial smoothing with Gaussian kernel of full width at half maximum 6 mm; high-pass temporal filtering with a filter width of 128 s (Gaussian-weighted least-squares straight line fitting, with sigma = 64.0 s); grand-mean intensity normalization of the entire 4D dataset by a single multiplicative factor; and individual level Independent Component Analysis denoising for motion and physiological noise using Multivariate Exploratory Linear Optimized Decomposition into Independent Components (version 3.15; Beckmann and Smith 2004), combined with an automated signal classifier (Tohka et al. 2008; Neyman-Pearson threshold = 0.3). For the spatial normalization, the EPI data were registered to the T1 image with a linear transformation, followed by a white-matter boundary-based transformation (Greve and Fischl 2009) using FLIRT, linear and nonlinear transformations to standard Montreal Neurological Institute (MNI) 2 mm brain were performed using Advanced Neuroimaging Tools (Avants et al. 2011), and then spatial normalization of the EPI image to the MNI. Quality check during preprocessing and analyses ensured adequate signal coverage.

### fMRI data analysis

For the Classmates Task, individual level, fixed-effects analyses were estimated using the general linear model convolved with a canonical hemodynamic response function using SPM12. The data were modeled as event-related using four separate conditions: high popularity, low popularity, peer acceptance, and peer rejection. We used the sociometric ratings within each condition as a parametric modulator at the trial level. Low popularity and peer rejection scores were originally negative (i.e. −1 SD below the mean) and high popularity and peer acceptance scores were positive (i.e. +1 SD above the mean). Because the raw sociometric scores were positive for accepted peers but negative for rejected peers, we took the absolute value, so that neural effects in each condition were comparable. In other words, the parametric modulator ranged from low scores to high scores in all conditions, which allowed us to test how neural activation increases as rejection/acceptance increases. For example, within the acceptance condition, the parametric modulator allows us to examine if brain regions show linear increases in BOLD signal as the absolute value of acceptance increases. Within the rejection condition, the parametric modulator allows us to examine if brain regions show linear increases in BOLD signal as the absolute value of rejection increases. Within each condition, the sociometric ratings were thus all positive, and ranged from low to high scores so we could examine how adolescents are tracking social status at the neural level. The repeated target within each block that served as an attention check was treated as a separate condition and modeled as a contrast of no interest. TRs with motion greater than 0.5 FD were modeled as a nuisance regressor.

From each individual’s first level models, we extracted parameter estimates of signal intensity from each of the ROIs (bilateral vmPFC, VS, and right TPJ). The ROIs were defined using different atlases built to highlight unique functional networks, a common analytical approach (McCormick et al. 2018). The right TPJ and bilateral vmPFC were defined using the Saxe Lab Theory of Mind ROIs (Dufour et al. 2013), and the bilateral VS was defined using the Harvard-Oxford Atlas (Harvard Center for Morphometric Analysis) at a 50% threshold.

We extracted parameter estimates from the ROIs for each condition of interest: accepted peers and rejected peers with the absolute value of the sociometric nomination as a parametric modulator at the trial level. Because of the parametric modulator, these parameter estimates of signal intensity represent BOLD signal that increases linearly with increases in peer acceptance or peer rejection. In the current study, we focus on neural responsivity to accepted and rejected peers for several reasons. First, prior studies link neurobiological sensitivity to peer rejection (versus acceptance) with more depressive symptoms (e.g. Platt et al. 2013). Second, rejection sensitivity plays a pivotal role in shaping perceptions of social support (e.g. Marston et al. 2010, Lazarus et al. 2016) as well as digital stress (Nick et al. 2022). Thirdly, social media make signals of rejection and acceptance highly tangible through features such as quantifiability and visual elements (Nesi et al. 2018). In this regard, both online rejection (e.g. cyber-ostracism) and online acceptance (e.g. feeling a sense of belonginess) have been linked to higher and lower depressive symptoms among adolescents, respectively (e.g. Smith et al. 2021, Ding et al. 2022). Combining adolescents’ heightened sensitivity to social status (Schriber and Guyer 2016) with the increased visibility of rejection/acceptance on social media, greater sensitivity to rejected or accepted peers may act as a neurobiological factor amplifying or mitigating the associations between social media and depression.

### Analysis plan

Hierarchical regression analyses were used to examine the associations between social media use (i.e. digital pressure and digital social connection on social media) and depressive symptoms at the 1-year follow up. VIF, Kurtoses, and Skewness values were examined to confirm that the models met linear assumptions. Bootstrapping was performed in all analyses and 95% confidence intervals are reported. The analyses controlled for baseline depressive symptoms, sex, and age at scan. Additionally, given the associations between acceptance/rejection and depressive symptoms among adolescents (e.g. He et al. 2018), and adolescents’ own social status may affect how their brains respond to peer acceptance/rejection cues (Zerubavel et al. 2015), participants’ sociometric scores (i.e. how much their peers accepted/rejected them) was controlled for as well. Lastly, in order to determine whether depressive symptoms are due to the nature of the experiences on these platforms or the sheer amount of time spent online, we also controlled for participants’ time spent on social media. Subsequently, we explored whether neural responsivity to peer acceptance/rejection in the vmPFC, VS, and rTPJ moderated the association between social media experiences (digital social connection, digital pressure) and depressive symptoms. Moderation was conducted by standardizing the predictor and moderator variables prior to calculating an interaction term. A hierarchical regression analysis was conducted whereby adolescents’ depressive symptoms (W4) served as the dependent variable, and baseline depressive symptoms, sex, age at scan, participants’ sociometric scores (i.e. how much their peers accepted/rejected them), frequency of social media use [We tested our analyses without frequency of social media use and participants’ sociometric social preference scores as covariates and results remained the same. Additionally, we also probed our models controlling for participants’ sociometric popularity score. Again, the results remained the same. Therefore, the models reported in the manuscript thus include baseline depressive symptoms, sex, age at scan, participants’ sociometric social preference scores (i.e. how much their peers accepted/rejected them), frequency of social media use, and parents’ reported SES (i.e. family annual income) as covariates], and parents’ reported Socio-Economic Status (i.e. SES measured by family annual income) were entered as covariates (Step 1), digital pressure and digital social connection were entered as predictors (Step 2), and the interaction term was added in the third step. We ran separate moderated regression analyses (in SPSS) for neural responsivity to accepted and rejected peers, within each ROI (i.e. a total of six analyses). For probing the significant moderation effects, we used the Johnson–Neyman technique and marginal-effects plots in conjunction with visual depictions of simple slope using small multiples (created with the R-based interActive data visualization tool; McCabe et al. 2018). To maintain statistical rigor, we implemented the Benjamini–Hochberg False Discovery Rate procedure, correcting for the total number of tests conducted (N_tests_ = 6).

## Results

Descriptive statistics and correlations between the variables of interest are shown in Table 1.

Additional test statistics comparing the means of males and females are shown in Table 2. Compared to males, females reported more depressive symptoms at W3 and W4 and reported more digital social connection as well as more digital pressure. There were no significant sex differences in neural responsivity to rejected and accepted peers in the ROIs.

**Table 2.** Zero-order correlations

	1	2	3	4	5	6	7	8	9	10	11	12	13	14	15
1.PSME3		0.45^**^	0.28*	0.08	0.24*	−0.01	−0.00	0.04	0.11	−0.02	0.19	0.25*	0.03	−0.12	0.05
2.DStress3			0.30^**^	0.21	0.01	0.05	−0.01	0.12	0.05	07	0.23*	0.24*	0.14	−0.30^**^	0.03
3.Depr3				0.47^**^	0.11	0.01	−0.01	0.18	−0.04	0.10	−0.02	0.32*	0.13	−0.10	−0.02
4.Depr4					−0.01	0.14	−0.17	0.26*	−0.05	0.23*	−0.07	0.41^**^	0.19	−0.07	−0.08
5.Acc_vmPFC						0.00	0.43^**^	0.13	0.50^**^	0.03	0.06	0.15	−0.21	−0.02	0.05
6.Rej_vmPFC							−0.02	0.46^**^	−0.13	0.62^**^	−0.07	0.04	−0.10	0.01	−0.13
7.Acc_rTPJ								0.13	0.49^**^	0.18	0.19	0.14	0.06	−0.06	−0.18
8.Rej_rTPJ									0.09	0.60^**^	0.20	0.16	−0.00	−0.07	−0.04
9.Acc_biVS										0.05	0.21	−0.08	−0.09	−0.18	0.00
10.Rej_biVS											0.12	0.18	0.10	−0.12	−0.13
11.SocPref												−0.00	0.02	−0.18	−0.26*
12.Sex													0.01	−0.02	−0.00
13.Age														−0.25*	0.06
14.FreqSM															

*Note*. PSME3, positive social media experiences at Wave 3; DStress3, digital pressure at Wave 3; Depr3, depressive symptoms at Wave 3; Depr4, depressive symptoms at Wave 4; Acc, neural responsivity to accepted peers; Rej, neural responsivity to rejected peers; SocPref, participants’ sociometric social preference at W2; FreqSM, frequency of social media use at W3.

* *P* < .05,

** *P* < .01.

Significant values are bolded.

### Longitudinal associations between social media use and depressive symptoms

To examine the longitudinal association between social media use and depressive symptoms, we first conducted a linear regression analysis. Results are displayed in Table 3. We did not find any significant associations between digital pressure, digital social connection and depressive symptoms, 1 year later.

**Table 3.** Means and standard deviations for all variables

	Overall sample			Female		Male		*P*
	M	SD	Range	M	SD	M	SD	
PSME3	2.98	1.15	1–5	3.25	1.04	2.68	1.21	.**02**
DStress3	1.96	0.96	1–5	2.16	0.94	1.72	0.93	.**04**
Depr3	0.38	0.47	0–2	0.52	0.53	0.22	0.32	**<.01**
Depr4	0.41	0.52	0–2	0.61	0.57	0.18	0.32	**<.001**
Acc_vmPFC	0.05	0.73	−1.83 to 2.23	0.15	0.68	−0.06	0.77	.19
Rej_vmPFC	−0.05	0.50	−1.88 to 1.13	−0.03	0.50	−0.07	0.50	.71
Acc_rTPJ	−0.04	0.45	−1.13 to 1.02	0.02	0.42	−0.10	0.49	.22
Rej_rTPJ	−0.04	0.44	−1.46 to 1.16	0.03	0.53	−0.12	0.32	.14
Acc_biVS	−0.06	0.50	−1.47 to 1.29	−0.09	0.47	−0.01	0.53	.48
Rej_biVS	−0.06	0.39	−1.26 to 0.86	0.01	0.40	−0.13	0.36	.10
SocPref	0.14	1.15	−3.92 to 3.57	0.31	1.04	−0.06	1.25	.15
Age	13.06	0.58	12–14	13.07	0.60	13.05	0.57	.90
FreqSM	3.11	2.38	1–9	2.98	2.31	3.27	2.48	.59
SES (Family annual income)	3.58	2.10	1–10	3.46	2.09	3.72	2.13	.59

*Note*. PSME3, positive social media experiences at Wave 3; DStress3, digital pressure at Wave 3; Depr3, depressive symptoms at Wave 3; Depr4, depressive symptoms at Wave 4; Acc, neural responsivity to accepted peers; Rej, neural responsivity to rejected peers; SocPref, participants’ sociometric social preference at W2; FreqSM, frequency of social media use at W3.

Means and standard deviations among individuals of different sexes for all the continuous variables are shown. The *t*-tests for sex were performed for each variable to test for group differences. Significant values are bolded.

### Moderating role of neural responsivity to accepted and rejected peers

To examine whether adolescents’ neural responsivity to accepted and rejected peers moderated links between digital pressure, digital social connection (W3) and depressive symptoms (W4), separate models were conducted for each ROI. Neural responsivity to rejected peers (i.e. when viewing peers whose social preference score was < 1 SD below the mean) and accepted peers (i.e. when viewing peers whose social preference score was >1 SD above the mean) was included as a moderator. Results are summarized in Table 4.

**Table 4.** Regression analysis linking digital pressure and positive experiences on social media to depressive symptoms at 1-year follow-up

Variable	∆R^2^	*β*	*P*	95% CI
Step 1—covariates	0.33	–	–	–
Sex	–	0.34	.002	0.13 to 0.58
Age	–	0.14	.17	−0.06 to 0.34
Frequency of social media use	–	0.00	.93	−0.04 to 0.05
Depressive symptoms T3	–	0.36	.001	0.16 to 0.62
SES (Family annual income)	–	−0.03	.77	−0.06 to 0.04
Social preference	–	−0.12	.25	−0.15 to 0.04
Step 2—main effect	0.02	–	–	–
Digital pressure	–	0.13	.43	−0.06 to 0.20
Positive experiences on social media	–	−0.15	.31	−0.17 to 0.04
Total *R*^2^	0.35	–	–	

*Note*. Durbin–Watson statistical value is 2.165 indicating no autocorrelation in the residuals from the regression analysis. –, data are not available.

For the association between digital pressure (W3) and depressive symptoms (W4), we did not find any significant moderations of neural responsivity to accepted or rejected peers within the vmPFC, rTPJ, and VS. No *post hoc* analyses were conducted. For the association between digital social connection (W3) and depressive symptoms (W4), we found significant moderations of neural responsivity to rejected but not accepted peers within the vmPFC, rTPJ, and VS (Table 4).

**Figure 1.**
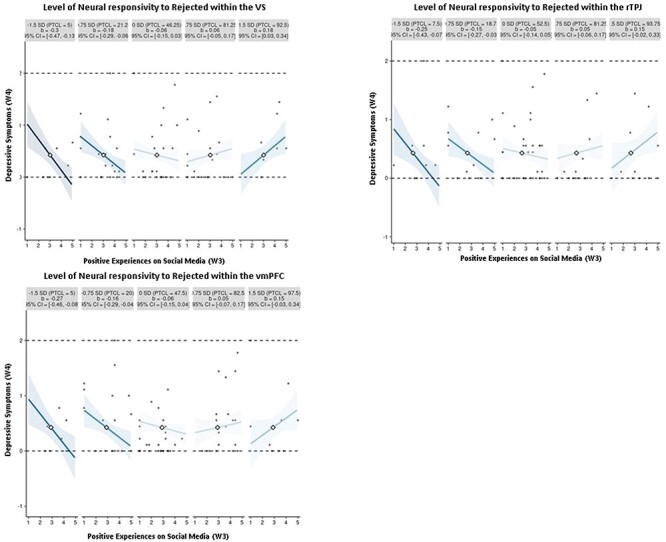
Small-multiples depictions of the interaction effect of positive experiences on social media and neural responsivity to rejected peers in the VS, rTPJ, and vmPFC.

*Note*. The small multiples display the interaction from 2 SD below to 2 SD above the mean, with each graphic showing the 95% confidence region (shaded), observed data (gray circles), outcome range (dashed lines), and crossover point (diamond), where z-transformed depressive symptoms indicate negative scores for below-average and positive for above-average depression.

We employed the Johnson–Neyman technique (McCabe et al. 2018) to probe these interactions. This method mathematically derives the regions of significance, indicating where the conditional effect of the predictor transitions from being statistically insignificant to statistically significant. We generated marginal effects plots (Fig. 1) using the interActive tool (McCabe et al. 2018).

The simple slope of digital social connection (W3) on adolescent depressive symptoms (W4) reaches significance under specific conditions. Specifically, when sensitivity to rejected peers within the VS is 0.25 SDs below the mean (accounting for 40% of observations), a significant negative association is observed. Conversely, when sensitivity to rejected peers within the VS is 1.25 SDs above the mean (comprising 7.5% of observations), depressive symptoms are higher when experiencing greater digital social connection. Additionally, the simple slope of digital social connection (W3) on adolescent depressive symptoms (W4) is significant and negative when neural activation toward rejected peers within the vmPFC is 0.35 SDs below the mean (with 30% of observations falling within the region of significance), but significantly positive when neural activation toward rejected peers is 2.2 SDs above the mean (with 1.25% of observations falling within this region of significance. Lastly, when neural activation toward rejected peers within the rTPJ is 0.45 SDs below the mean (23.75% of observations), a significant negative association is observed. Conversely, when neural activation toward rejected peers within the rTPJ is 2 SDs above the mean (comprising 2.5% of observations), depressive symptoms are higher when experiencing greater digital social connection.

**Figure 2.**
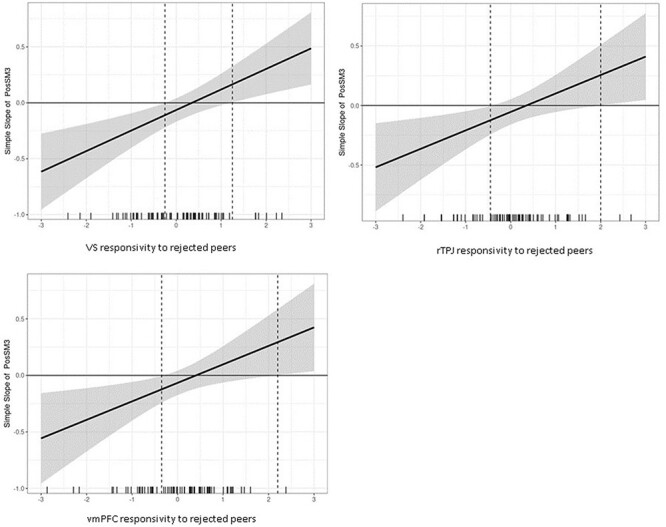
Conditional values of neural responsivity to rejected peers in VS, vmPFC, and rTPJ by values of the simples slope of 1-year depressive symptoms regressed onto positive experiences on social media.

*Note*. Simple slopes outside the dotted lines are significant, with the gray area representing confidence intervals across all conditional values of the moderator (VS).

For visualization purposes, we further used small multiples to plot a broad range of simple slope effects, showcasing data points that are most representative of each simple slope (McCabe et al. 2018). As shown in Fig. 2, among adolescents with lower sensitivity to rejection within the VS, rTPJ, and vmPFC (i.e. the two leftmost plots at −1.5 SDs and −0.75 SDs), digital social connection was associated with less depressive symptoms at the 1-year follow-up. Conversely, individuals with relatively moderate sensitivity in the VS, rTPJ, and vmPFC (i.e. the middle graphs at 0 SD and 0.75 SDs) showed no discernible link between digital social connection and depressive symptoms 1 year later. For those with increasing sensitivity to rejection within the VS (i.e. positive values at 1.5 SDs above the mean; rightmost plot), the association reversed, indicating that digital social connection was related to increased levels of depressive symptoms for adolescents with high neural responsivity in the VS.

## Discussion

Social media platforms have been presented as a double-edged sword (Choukas-Bradley et al. 2023), offering adolescents opportunities for positive social connection while also potentially exposing them to stress due to the social expectation of constant online presence. Our findings underscore the importance of considering individual differences, and neurobiological sensitivities to rejected peers in particular, in determining which adolescents may benefit or suffer most from their (positive) social media experiences.

### Diverse social media experiences and depressive symptoms

Building upon previous research that connects social media use with adolescents’ mental well-being in both positive and negative directions (e.g. Orben and Przybylski 2019), our study aligns with recent attempts (Nesi et al. 2022) to distinguish between various online experiences and adolescents’ emotional responses. Specifically, delving into the distinct nature of online interactions is a step toward identifying the mechanisms through which social media use can present both opportunities and risks for adolescent well-being. Interestingly, we did not find a longitudinal relationship between digital social connection or digital pressure and depressive symptoms at 1-year follow-up (H1 and 2). Given that we distinguished between the types of online experiences, we expected to find associations with adolescents’ depressive symptoms. Nonetheless, this is consistent with a growing body of work finding null or weak associations between social media experiences and adolescents’ well-being (e.g. Beyens et al. 2021). Some scholars have argued that not all adolescents will show a (positive nor negative) link between their social media use and well-being and that looking at a sample as a whole might lead to null results because it negates the possibility of individual differences (e.g. Valkenburg et al. 2021).

Notably, applying the framework of the differential susceptibility model, we examined whether individuals who were more neurobiologically sensitive to their rejected and accepted peers in brain regions involved in valuation and social cognition (i.e. the vmPFC, VS, and rTPJ) would show a stronger association between their social media experiences and subsequent depressive symptoms. In this context, more sensitive adolescents were hypothesized to thrive in a positive online environment but be more vulnerable in a negative one, while those with lower sensitivity would be less affected by both digital social connection and digital pressure (H3 and H4).

### Low neural responsivity to rejected peers, digital social connection, and depressive symptoms

We found that those who were less sensitive to their rejected peers (i.e. showing decreasing levels of activation in rTPJ, vmPFC, and VS when exposed to increasingly rejected peers), experienced fewer depressive symptoms when they reported greater digital social connection. These findings suggest that a diminished neural response to rejection may function as a protective factor against the development of depressive symptoms, when these adolescents have positive online interactions.

Adolescents often use mentalizing as a crucial tool for navigating intricate social environments, such as peer social hierarchies (Brown and Larson 2009). This skill becomes especially vital in digital environments, where social interactions offer fewer interpersonal cues and where indicators of social status are prominently on display (Nesi et al. 2018). The TPJ is a key region involved in mentalizing. Decreasing sensitivity in the TPJ in response to rejected peers was related to decreased depressive symptoms in the context of digital social connection, suggesting that adolescents with dampened TPJ sensitivity appeared to thrive the most in a positive online context. It is possible that adolescents with lower rTPJ sensitivity to their rejected peers may have reduced sensitivity to the distress of others, which could potentially enable these adolescents to experience less distress in response to their own social media interactions and even thrive in positive social media contexts.

**Table 5.** Neural responsivity to social preference moderates associations between positive experiences on social media and depressive symptoms, at 1-year follow-up

	Depressive symptoms (T4)				
	*b* [95% CI]	SE	β	*P*	Adjusted *P*
Positive experiences on social media					
vmPFC					
Accepted*PSME	−0.04 [−.18, 0.10]	0.07	−0.06	.59	.71
Rejected * PSME	**0.27 [0.06, 0.49]**	**0.11**	**0.26**	**.01**	**.003**
rTPJ					
Accepted*PSME	0.01 [−.21, 0.24]	0.11	0.01	.92	.92
Rejected*PSME	**0.30 [0.07, 0.52]**	**0.11**	**0.26**	**.01**	**.026**
biVS					
Accepted*PSME	0.06 [−.12, 0.23]	0.09	0.07	.51	.76
Rejected*PSME	**0.41 [0.18, 0.64]**	**0.11**	**0.35**	**<.001**	**<.001**
Digital pressure					
vmPFC					
Accepted*DS	−0.10 [−.28, 0.08]	0.09	−0.12	.27	.54
Rejected* DS	0.08 [−.10, 0.27]	0.09	0.09	.38	.45
rTPJ					
Accepted*DS	−0.15 [−.37, 0.08]	0.11	−0.12	.20	.61
Rejected*DS	0.12 [−.10, 0.34]	0.11	0.11	.30	.45
biVS					
Accepted*DS	−0.04 [−.22, 0.14]	0.09	−0.04	.68	.68
Rejected*DS	0.25 [−.00, 0.49]	0.12	0.19	.05	.31

*Note*. Participants’ sex, sociometric social preference, age, frequency of social media use, and baseline depressive symptoms were controlled for in the models. *P*-values were corrected using the Benjamini–Hochberg False Discovery Rate procedure for multiple comparisons. Significant values are bolded.

The study’s findings further suggest an intriguing relationship between brain regions associated with reward processing and the experience of depressive symptoms in the context of online interactions. We found that a decrease in sensitivity in both the VS and vmPFC when viewing highly rejected peers was associated with reduced depressive symptoms in the context of positive social media experiences. The vmPFC, recognized for its involvement in both negative affect and reward processing, is thought to play a multifaceted role in emotion regulation, including fear-response inhibition and emotional response modulation, as suggested by previous fMRI studies in fear conditioning (Fullana et al. 2016, Doré et al. 2017). The VS is further implicated in reward learning (Daniel and Pollmann 2014). Our findings of decreasing sensitivity of the vmPFC and VS when viewing pictures of rejected peers seems to align with the idea that the vmPFC and VS may serve as a pathway for translating newly generated conceptual information into affective behavioral and physiological responses, which can either strengthen or diminish salience signaling (Andrewes and Jenkins 2019, Steward et al. 2021). Therefore, decreasing sensitivity in the vmPFC and VS in response to images of rejected peers may suggest a dampened salience of rejected peers, which may serve a protective function. This may help adolescents to savor feelings of online connection and lead to a decrease in depressive symptoms.

### High neural responsivity to rejected peers, digital social connection, and depressive symptoms

Our findings further revealed that greater digital social connection (at Wave 3) related to elevated depressive symptoms (at Wave 4) for those showing increased activation in the rTPJ and VS in response to images of rejected peers. Perhaps, those with heightened neural responsivity to rejected peers may disproportionately process and respond to positive aspects of the social environment (Schriber and Guyer 2016), including the online context. Negative processing biases (Platt et al. 2013) could hinder these adolescents from fully embracing and savoring positive online experiences by, for instance, fostering a predisposition to interpret even favorable interactions in a negative light.

Individuals with increased rTPJ and VS activation in response to rejection cues may exhibit heightened sensitivity and selective attention to cues associated with social rejection (Schriber and Guyer 2016). In the context of digital social connection, this heightened sensitivity may then translate into a focus on (potentially subtle) signs of rejection or it could diminish the rewarding aspects of positive online interactions. As such, individuals might be hindered in deriving satisfaction and pleasure from these positive experiences, which contributes to depressive symptoms.

Higher-level cognitive biases, such as attributional style and negative expectations, have been shown to heighten depressive responses to psychosocial stress (Platt et al. 2013). Our findings seem to extend this notion, suggesting that these biases may manifest even in a positive online context. Nevertheless, a note of caution is necessary in the interpretation of this result and future research is needed to scrutinize these assumptions more thoroughly.

### Digital pressure

Contrary to our expectations, neurobiological sensitivity to accepted/rejected peers did not moderate the examined associations between digital pressure on social media and depressive symptoms. One possible explanation might be that stressful situations elicit more activation. Specifically, prior research has shown that youth exhibit stronger reactivity in response to peer rejection compared to acceptance (Silk et al. 2012). Additionally, there were no significant moderations of neurobiological sensitivity in the context of digital pressure. Potentially, digital pressure might be influenced by a multitude of factors beyond sensitivity to social evaluation, making it a more complex construct to study. For instance, research has shown how digital pressure might be influenced by the dynamics within specific friendship cliques (De Groote and Van Ouytsel 2022). Therefore, understanding the role of social norms and expectations on digital availability might be fruitful to include.

### Limitations and future directions

The current study has several strengths including its longitudinal design and use of an ecologically valid fMRI task to examine adolescents’ neural responsivity to their real life rejected and accepted peers. However, several limitations should be acknowledged. First, the study relied on self-report measures of social media experiences and emotional responses to those experiences. Second, the fMRI task was completed 1 year prior to the longitudinal data collection (Waves 3 and 4). Although brain activation may change over time, our examination of neural responsivity as a trait index aligns with the biological sensitivity to context theories (Belsky et al. 2007, Ellis and Boyce 2008). Therefore, the temporal gap does not significantly undermine the validity of our findings. Still, future studies should endeavor to reduce the time gap between neural assessments and behavioral data collection to further validate and refine these findings. Further, the analytical sample (*N* = 80) might have limited the generalizability of the current findings and future studies employing larger sample sizes are advised to show consistent brain–behavior associations (e.g. Marek 2022).

For future directions, studies might want to explore both depressive symptoms and healthy psychological adjustment to capture the multifaceted nature of adolescents’ engagement with social media and its impact on their overall well-being. Additionally, future studies might benefit from including emotion regulation processes as an explanatory factor for the examined effects. Specifically, individual differences in susceptibility might influence an individual’s capacity for emotion regulation. For instance, one study found that in girls who were highly sensitive to social rejection and had a history of increased peer victimization, there was evidence of less effective neural regulation of emotion (i.e. positive amygdala–rVLPFC connectivity) (Rudolph et al. 2021). As a result, it could be important to explore whether rejection sensitivity limits individuals’ capacity to regulate their emotions when exposed to various online experiences, whether positive or negative, and whether this could have a detrimental effect on their well-being.

## Conclusion

The pervasive presence of social media in the lives of adolescents can carry substantial implications for their mental well-being, for better or worse. The findings of this study suggest that individual differences in neural responsivity to rejected peers might play a role as a risk (high neural responsivity) or protective (low neural responsivity) factor in the link between social media use and depressive symptoms. The results underscore that some adolescents might be more likely to or capable of savoring online connection (i.e. positive online experiences) than others.
